# Child-Pugh Parameters and Platelet Count as an Alternative to ICG Test for Assessing Liver Function for Major Hepatectomy

**DOI:** 10.1155/2017/2948030

**Published:** 2017-08-29

**Authors:** Kin-Pan Au, See-Ching Chan, Kenneth Siu-Ho Chok, Albert Chi-Yan Chan, Tan-To Cheung, Kelvin Kwok-Chai Ng, Chung-Mau Lo

**Affiliations:** Department of Surgery, The University of Hong Kong, Hong Kong

## Abstract

**Objective:**

To study the correlations and discrepancies between Child-Pugh system and indocyanine green (ICG) clearance test in assessing liver function reserve and explore the possibility of combining two systems to gain an overall liver function assessment.

**Design:**

Retrospective analysis of 2832 hepatocellular carcinoma (HCC) patients graded as Child-Pugh A and Child-Pugh B with ICG clearance test being performed was conducted.

**Results:**

ICG retention rate at 15 minutes (ICG15) correlates with Child-Pugh score, however, with a large variance. Platelet count improves the correlation between Child-Pugh score and ICG15. ICG15 can be estimated using the following regression formula: estimated ICG15 (eICG15) = 45.1 + 0.435 × bilirubin − 0.917 × albumin + 0.491 × prothrombin time − 0.0283 × platelet (*R*^2^ = 0.455). Patients with eICG15 >20.0% who underwent major hepatectomy had a tendency towards more posthepatectomy liver failure (4.1% versus 8.0%, *p* = 0.09) and higher in-hospital mortality (3.7% versus 8.0%, *p* = 0.052). They also had shorter median overall survival (5.10 ± 0.553 versus 3.01 ± 0.878 years, *p* = 0.015) and disease-free survival (1.37 ± 0.215 versus 0.707 ± 0.183 years, *p* = 0.018).

**Conclusion:**

eICG15 can be predicted from Child-Pugh parameters and platelet count. eICG15 correlates with in-hospital mortality after major hepatectomy and predicts long-term survival.

## 1. Introduction

Pugh's [1] gut feeling prompted the modification of the classification devised by Child [2] in the assessment of liver function reserve. Child-Pugh's [3] classification has stood the test of time in guiding treatment decisions and prognostication of patients with hepatocellular carcinoma (HCC) in the west [4]. The five variables are bilirubin, albumin, prothrombin time, ascites, and presence of hepatic encephalopathy. Application of these five factors was the judgment made by very experienced clinicians. It was subsequently validated by numerous clinical series in a recursive manner [5, 6]. Excretory function of the liver is expressed by the serum bilirubin level. Synthetic functions are assessed by the serum albumin level and prothrombin time. Detoxification failure leads to hepatic encephalopathy. Development of ascites is a consequence of poor synthetic function and portal hypertension. Child-Pugh A and certain Child-Pugh B patients are candidates for major hepatectomy [7].

Quantitative assessment by indocyanine green (ICG) clearance test is more commonly used in the East [8]. ICG is a water-soluble fluorescent cyanine dye. In vivo it is exclusively excreted by the liver into the bile without metabolism or enterohepatic circulation [9]. The ICG retention is an indirect assessment of functional hepatic blood flow [10]. Cirrhotic liver decreases hepatic blood flow and results in an increase in total splanchnic sequestration [11]. In the trauma model, it is used to assess splanchnic haemodynamics [12]. An ICG retention rate at 15 minutes (ICG15) of no more than 14.0% has been validated as a minimal requirement for major hepatectomy [13], as defined by resection of 4 or more Couinaud segments [14]. ICG15 is however not used in most centres in the west [15], and reliance is rather on Child-Pugh score and grade [7].

Though ICG clearance test is a step further to assess liver functional reserve, it is an additional test. The facilities to measure ICG15 are not readily available in some centres. In this study, we looked into the correlation between ICG retention rate with Child-Pugh score and grade in a consecutive series of patients in our centre. Discrepancy between the two systems is just as important. The aim was to better apply the two systems and to explore the possibility of combining and complementing the advantages of the two systems. In our study, the unique role of platelet count in associating Child-Pugh score and ICG15 has been highlighted. Thrombocytopenia is a constant phenomenon of portal hypertension [16]. Clinically, it is associated with increased posthepatectomy liver failure and operative mortality [17]. However, it has not been routinely considered during preoperative liver function assessment. In this study, we investigated the additional role of platelet count when combined with preexisting liver function assessment.

## 2. Methods

### 2.1. Patients

**Figure 1:**
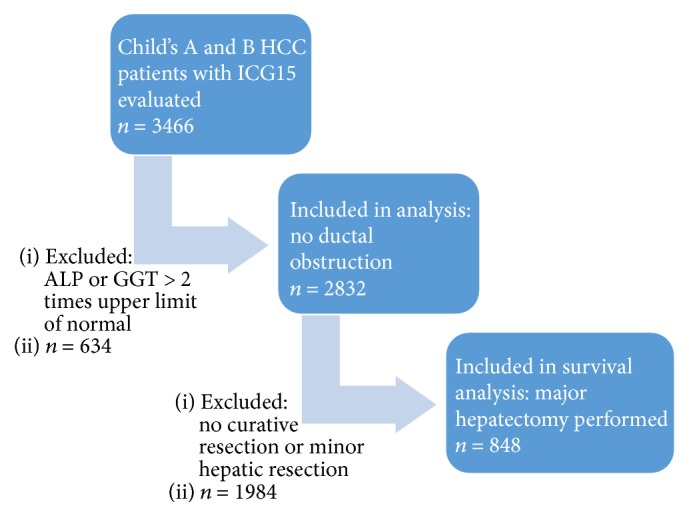
Flow diagram of this study.

From May 1989 to December 2015, 3466 HCC patients graded A or B by Child-Pugh system underwent ICG clearance test to evaluate for liver resection in the Department of Surgery, Queen Mary Hospital, the University of Hong Kong. 634 patients with alkaline phosphatase (ALP) or gamma-glutamyl transferase (GGT) levels higher than 2 times upper limit of normal were excluded, as their ICG15 could have been affected by ductal obstruction. The remaining 2832 patients formed the basis of this retrospective study. 848 patients who underwent major hepatectomy were included for survival analysis. The number of patients analysed in each step of this study was summarized in Figure 1.

All patients were regularly followed up at our outpatient clinic and were prospectively monitored for recurrence by serum alpha-fetoprotein level and contrast computed tomography (CT) scan, together with chest X-ray, every 3 to 6 months. A computerized database has been established since 1989 for prospective collection of patient data. Any postoperative recurrence was entered into the database immediately upon diagnosis.

### 2.2. ICG Clearance Test

ICG clearance test was performed by rapid intravenous injection of 0.5 mg/kg body weight of ICG. After a 15-minute interval, blood was drawn from the contralateral cubital vein and read with a spectrophotometer. ICG retention was expressed as a percentage of the fluorescent dye retained at 15 minutes.

### 2.3. Statistics

Factors affecting ICG15 were identified with univariate and multivariate linear regressions. ICG15 was plotted against Child-Pugh score with their standard deviations denoted by error bars. Patients who received surgical resection were further analysed for survival with Kaplan-Meier curve and compared with log-rank test. Their baseline characteristics were compared, with *t*-test for continuous variables and chi-square test for discrete variables. Statistic calculations were computed with Statistical Product and Service Solution (SPSS) 16.0.

## 3. Results

### 3.1. Relation between ICG15 and Child-Pugh Score

**Figure 2:**
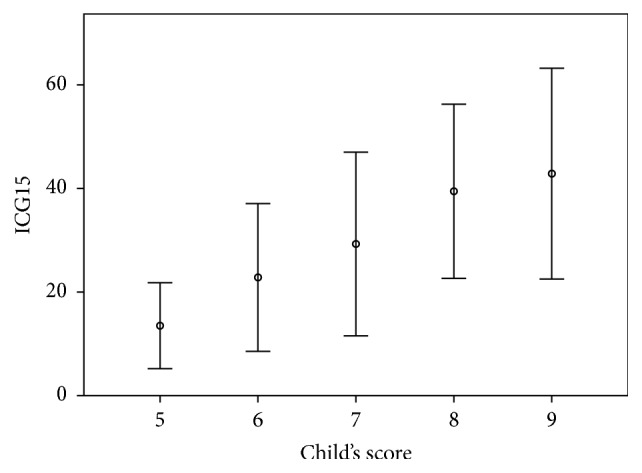
Correlation between ICG15 and Child-Pugh score. A linear relationship is expressed by ICG15 = −27.4 + 8.21 × Child-Pugh score (*R*^2^ = 0.264). Error bars: ±1 SD.

There is an almost linear correlation between ICG15 and the Child-Pugh score (Figure 2). The low *R*^2^ value (0.264) of this linear relationship and the large deviance of ICG15 (standard deviation 8.30–20.3) reflect heterogeneity among patients with same Child-Pugh score. This is of great importance, as patients with prolonged ICG retention are at risk of liver failure after major hepatectomy. Using local ablative therapy extensively for multiple lesions may result in hepatic dysfunction.

### 3.2. Factors Affecting ICG15

The Child-Pugh score comprises fiver parameters: ascites, hepatic encephalopathy, bilirubin, albumin, and prothrombin time. Linear regression analysis (Table 1) shows that bilirubin level (OR 1.63, 95% CI 1.49–1.78, *p* < 0.001), albumin level (OR 0.461, 95% CI 0.381–0.556, *p* < 0.001), and prothrombin time (OR 2.28, 95% CI 1.18–4.40, *p* = 0.014) predict ICG15. Ascites and grading of hepatic encephalopathy do not affect ICG clearance rate. Liver enzymes including alanine aminotransferase (ALT) and gamma-glutamyl transferase (GGT) do not affect ICG15.

**Figure 3:**
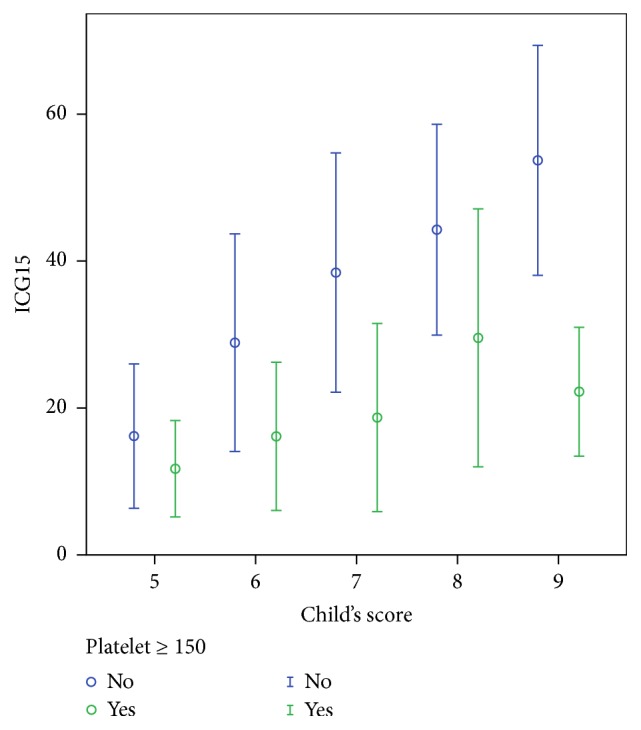
Correlation between ICG15 and Child-Pugh score stratified by platelet count. Error bars: ±1 SD.

Thrombocytopenia is a constant phenomenon of portal hypertension [16]. Low platelet count is associated with increased posthepatectomy liver failure and operative mortality [17]. The question of this being a confounding factor of the above five, or an additional parameter to assess liver function reserve, is worth investigating. In our series, platelet count remains a robust independent predictor of ICG15 (OR 0.973, 95% CI 0.963–0.983, *p* < 0.001). In Table 2, when patients in each category of Child-Pugh score are subdivided into two groups based on the platelet count equal to or above 150 × 10^9^/L, that is, normal and those below normal, the ICG15 of the patients in this series diverted in a predictable manner. As shown in Figure 3, incorporation of platelet count improves the correlation between ICG15 and Child-Pugh score. Patients with thrombocytopenia consistently have slower ICG clearance across all Child-Pugh groups. It is worth noting that patients with normal platelet count constantly outperform their counterparts with lower Child-Pugh scores. For example, Child-Pugh score 7 patients with normal platelet count have better ICG15 (18.7 ± 12.8) than Child-Pugh score 6 patients with thrombocytopenia (28.9 ± 14.8). Child-Pugh score 8 patients with normal platelet count have lower ICG15 (29.5 ± 17.6) as those who scored 7 but with thrombocytopenia (38.4 ± 16.3) (Table 2 and Figure 3). This highlighted the role of platelet count in correlating the two systems.

Table 3 summarizes the differences between patients with normal and low platelet counts. Minimal numbers of hepatic encephalopathy [2 (0.125%) versus 3 (0.244%), *p* = 0.66] were found in both groups, of which its presence would preclude surgical treatment in most situations. As expected, patients with normal platelet count had fewer numbers of ascites [66 (4.12%) versus 78 (6.34%), *p* = 0.001], as both thrombocytopenia and ascites result from portal hypertension. Patients with normal platelet count also have lower level of bilirubin (12.8 ± 7.67 versus 17.7 ± 11.4 *µ*mol/L, *p* < 0.001), higher level of albumin (40.0 ± 5.03 versus 38.4 ± 5.28 g/L, *p* < 0.001), and less prolonged prothrombin time (12.3 ± 1.65 versus 13.1 ± 1.65, *p* < 0.001). These differences, though statistically significant, were hardly clinically evident. By contrast, the normal platelet group performed remarkably better in terms of ICG clearance (ICG15 13.0 ± 8.29 versus 21.9 ± 14.9%, *p* < 0.001). This reemphasizes the unique importance of platelet count as the missing link between Child-Pugh score and ICG retention test.

### 3.3. Prediction of ICG15 from Child-Pugh Parameters and Platelet Count

We investigated the possibility of estimating ICG15 from objective Child-Pugh parameters and platelet count. Ascites and hepatic encephalopathy are not included for analysis, because clinical assessment of their grading could be subjective and because of the paucity of positive samples. In our series, numbers of patients with ascites or hepatic encephalopathy were few; therefore, their inclusion is unlikely to yield meaningful results.

By stepwise multiple linear regression analysis, the relation between ICG15 with bilirubin, albumin, prothrombin time, and platelet count was tested. The following formula is derived:

(1) Estimated  ICG15 eICG15=45.1+0.435×Bilirubin−0.917×Albumin+0.491×Prothrombin  time−0.0283×PlateletR2=0.455.

**Figure 4:**
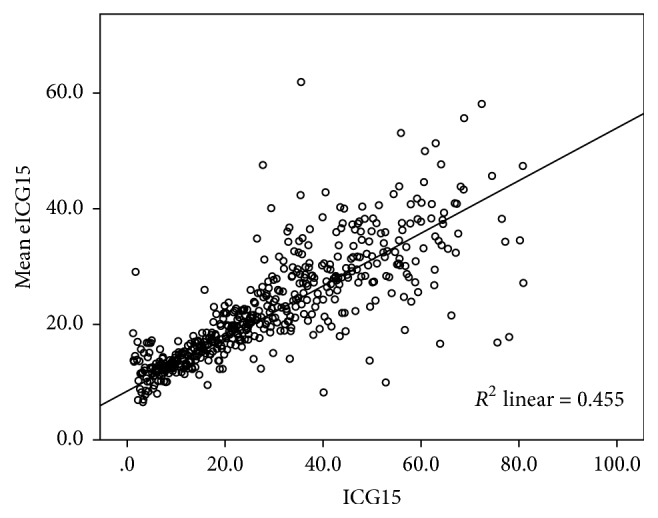
Relation between mean eICG15 and ICG15.

**Figure 5:**
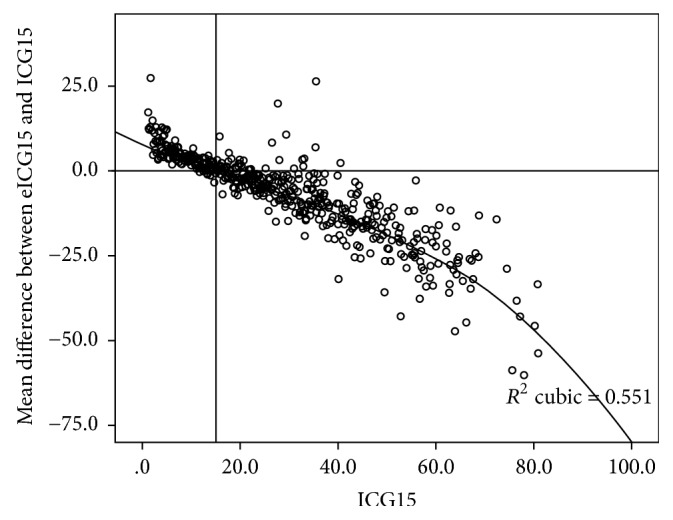
Mean error of eICG15 against ICG15.

Mean eICG15 from this formula was plotted against ICG15 on Figure 4. The linear correlation is stronger while ICG15 is relatively small, that is, less than 30%.  Figure 5 plotted the mean error of this estimation against measured ICG15. The formula performs best when ICG15 is 15%, close to the value of 14% which is of clinical interest. Estimation becomes less accurate when ICG15 is large, that is, higher than 30%.

**Figure 6:**
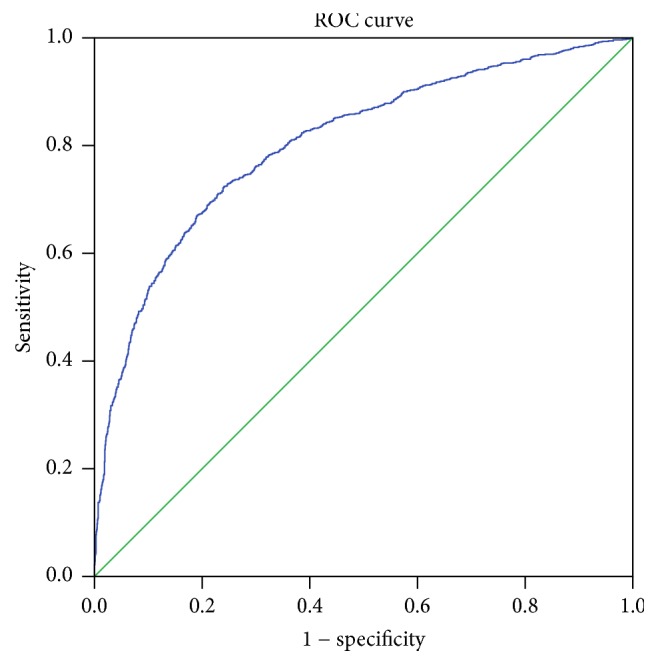
Receiver operating characteristic curve determining cut-off point of eICG15 to predict ICG15 ≤14%. Area under curve = 0.804. Diagonal segments are produced by ties.

**Figure 7:**
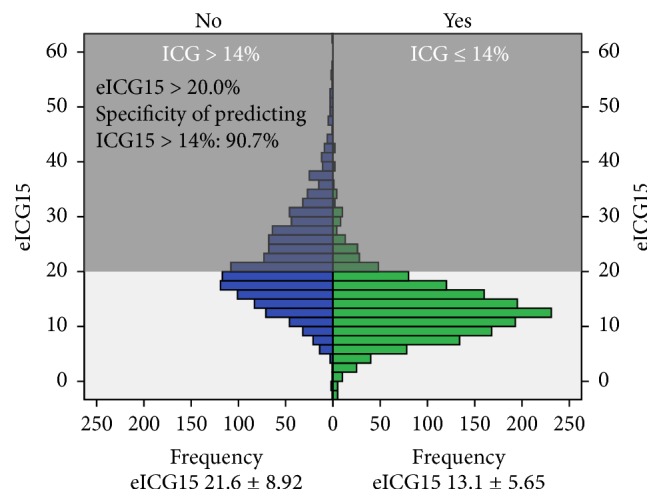
Distribution of patients with ICG15 >14% by eICG15.

Receiver operating characteristic curve (Figure 6, area under curve = 0.804) shows that a cut-off of eICG15 >20.0% predicts ICG15 >14.0% by a specificity of 90.7%. Numbers of patients with measured ICG15 ≤14% are plotted against eICG15 in Figure 7. Patients in the shaded area have an eICG15 higher than 20.0%, and among them less than 9.3% would have an ICG15 less than or equal to 14%, that is, agreeable to major hepatectomy.

### 3.4. Clinical Implications of eICG15

In our series, 848 patients underwent major hepatectomy. 761 (89.7%) of them had an eICG15 ≤20.0%, while 87 (10.3%) of them had a level higher than that (Table 4). Patients with eICG15 ≤20.0% had clinically observable differences in terms of Child-Pugh parameters. They had lower level of bilirubin (10.9 ± 4.45 versus 20.9 ± 12.2 *µ*mol/L, *p* < 0.001), higher level of albumin (41.2 ± 3.95 versus 34.1 ± 4.40 g/L, *p* < 0.001), and less prolonged prothrombin time (12.1 ± 1.16 versus 13.3 ± 2.44, *p* < 0.001). They also had higher platelet counts (209 ± 81 versus 175 ± 67, *p* < 0.001).

There is a trend towards more posthepatectomy liver failure in patients with eICG15 >20.0% [31 (4.1%) versus 7 (8.0%), *p* = 0.09]. eICG15 > 20.0% also predicted higher in-hospital mortality after major hepatectomy [28 (3.7%) versus 7 (8.0%), *p* = 0.052].

**Figure 8:**
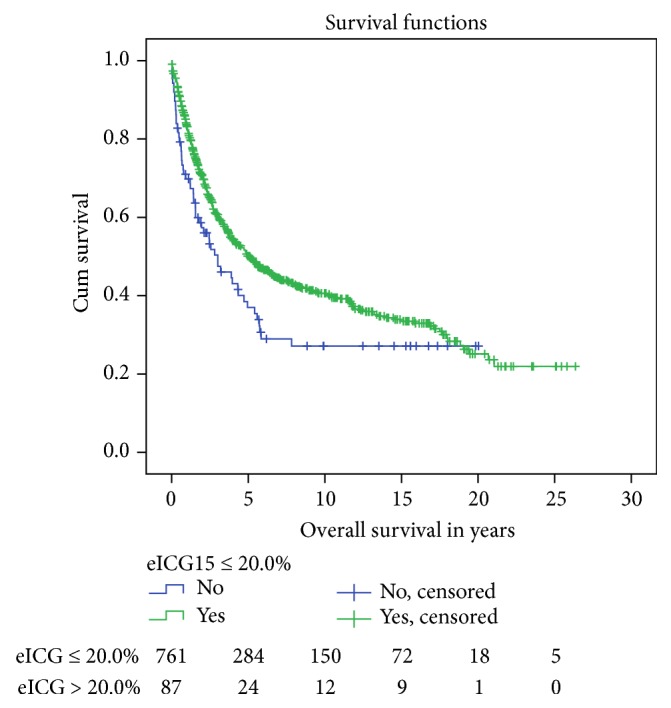
Overall survival after major hepatectomy, stratified by eICG15. Median overall survival 5.10 ± 0.553 versus 3.01 ± 0.878 years, *p* = 0.015.

**Figure 9:**
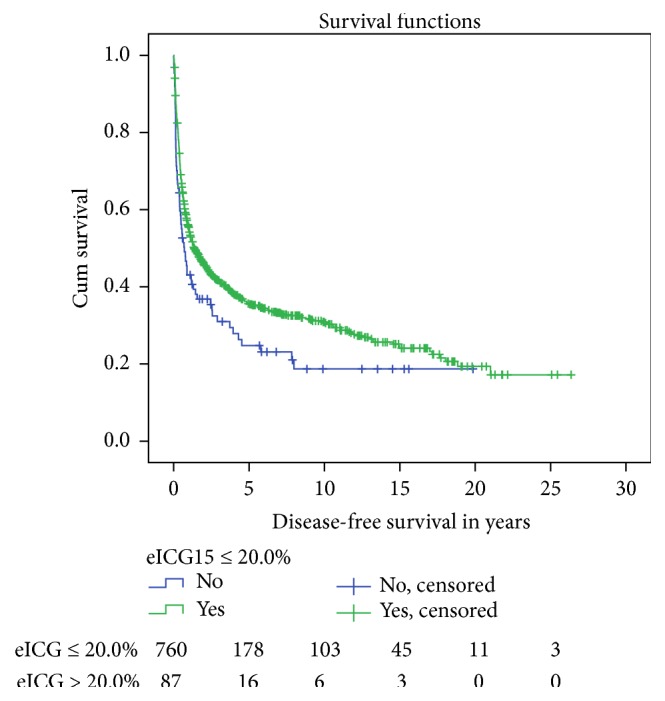
Disease-free survival after major hepatectomy, stratified by eICG15. Median disease-free survival 1.37 ± 0.215 versus 0.707 ± 0.183 years, *p* = 0.018.

In long term, eICG15 > 20.0% indicated inferior oncological outcomes. Both groups of patients have comparable tumour size (6.97 ± 3.72 versus 8.23 ± 4.42 cm, *p* = 0.13). Despite having fewer tumours with portal venous invasion (5.3% versus 1.5%, *p* = 0.04), patients with eICG15 >20% had shorter median overall survival (5.10 ± 0.553 versus 3.01 ± 0.878 years, *p* = 0.015) (Figure 8) and disease-free survival (1.37 ± 0.215 versus 0.707 ± 0.183 years, *p* = 0.018) (Figure 9).

## 4. Discussion

**Figure 10:**
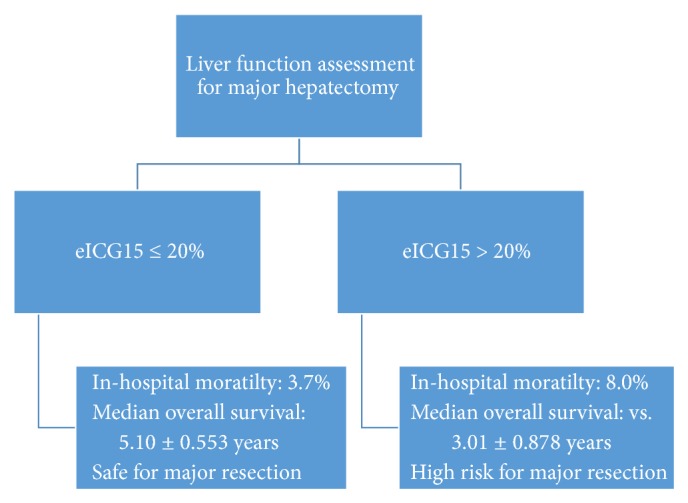
Algorithm for interpreting eICG15.

Assessing the degree of functional impairment of the liver from cirrhosis is pivotal to the choice of treatment of HCC, as various treatment modalities inflict various extent of insult to the cirrhotic liver. The advantage of the Child-Pugh score and grade is that a comprehensive assessment of liver function could be readily determined from routine blood tests and careful clinical assessment. ICG15 provides a more gradated evaluation over the ordinal scale, however, at the expense of additional invasiveness and cost. ICG contains sodium iodide and ICG clearance test is contraindicated for patients allergic to iodide. Adverse reactions occur in 0.7% of patients receiving intravenous ICG injection [18]. Additional labour and equipment, that is, spectrometer, are required. Platelet count improves the correlation between the two systems (Figure 3). It is constantly an important parameter in assessing liver function [16] and reflects the prognosis of HCC patients [19, 20]. Our regression formula combines Child-Pugh parameters and platelet count to achieve a quantitative assessment as with ICG15, so that the simplicity of Child-Pugh system and the agility of ICG15 are incorporated. Using a cut-off value of 20.0%, eICG15 identifies patients with higher operative risk (in-hospital mortality after major hepatectomy 3.7% versus 8.0%, *p* = 0.052) and inferior oncological outcomes (median overall survival 5.10 ± 0.553 versus 3.01 ± 0.878 years, *p* = 0.015). These figures should be considered before major hepatectomy is offered (Figure 10).

The standard deviation of ICG when plotted against Child-Pugh score was large (8.30–20.3). Thus, whether Child-Pugh score may replace ICG15 is questioned. Correlation is improved when patients were stratified by their platelet counts (Figure 3). The fact that patients with normal platelet have better ICG15 compared to their counterparts with lower Child-Pugh scores indicates the necessity of taking platelet count into consideration when relating the two systems. Using platelet count and objective Child-Pugh parameters, our regression formula gives an equally simple yet more gradated estimation of liver function reserve comparing to Child-Pugh score. The formula provides reliable estimation when the ICG15 is closed to the clinically interested value of 14% (Figure 5).

Though the Child-Pugh system and ICG clearance test have been employed in parallel in the west and the East for a quarter of century, literature describing their relation was scarce. This study delineates the correlation and discrepancies between them. We are first to emphasize the role of platelet count in associating the two systems and to correlate both using a regression formula. The implications of this formula have been emphasized by its prediction of both operative and oncological outcomes. The greatest limitation of this study is its retrospective nature. Further validation of this formula would be with prospective data that would allow us to overcome this weakness. ICG clearance test was performed for potential surgical candidates and patients who were obviously not candidate for surgery; that is, Child-Pugh C liver function were not included. When the subset of operated patients was compared stratified by their eICG15, the two groups differed in terms of extent of surgery. Nevertheless, the sample in this series resembled the actual patients of which surgery is contemplated. This study presents a simple liver function reserve assessment to guide clinicians in treatment decision and prognosis for HCC patients.

## Figures and Tables

**Table 1:** Univariate and multivariate analysis for factors associated with ICG15.

	Univariate analysis		Multivariate analysis	
	OR (95% CI)	*p*-value	OR (95% CI)	*p* value
Ascites	16 (804–14191)	<0.001	0.98 (0.0660–14.4)	0.99
HE	10.3 (0.0433–2453)	0.40		
Bilirubin	1.90 (1.82–1.98)	<0.001	1.63 (1.49–1.78)	<0.001
ALT/SGPT	1.02 (1.02–1.03)	<0.001	1.01 (0.987–1.03)	0.53
GGT	1.04 (1.00–1.08)	0.038	1.02 (0.993–1.05)	0.15
Albumin	0.298 (0.276–0.321)	<0.001	0.461 (0.381–0.556)	<0.001
PT	12.9 (10.0–16.7)	<0.001	2.28 (1.18–4.40)	0.014
Platelet	0.956 (0.951–0.960)	<0.001	0.973 (0.963–0.983)	<0.001
Heamoglobin	0.28 (0.221–0.355)	<0.001	0.720 (0.439–1.18)	0.19

**Table 2:** ICG15 of patients with various Child-Pugh score stratified by platelet count.

Child-Pugh		Platelet ≥ 150 × 10^9^/L				Platelet < 150 × 10^9^/L			
		ICG15 (mean ± SD)		Number/%		ICG15 (mean ± SD)		Number/%	
5	A	11.7 ± 6.57	12.4 ± 7.39	1264	44.6%	16.2 ± 9.81	19.2 ± 12.4	827	29.2%
6		16.1 ± 10.1		231	8.16%	28.9 ± 14.8		254	8.97%

7	B	18.7 ± 12.8	21.0 ± 14.0	77	2.72%	38.4 ± 16.3	42.0 ± 16.4	89	3.14%
8		29.5 ± 17.6		20	0.71%	44.3 ± 14.3		40	1.41%
9		22.2 ± 8.78		10	0.35%	53.7 ± 15.6		20	0.71%

**Table 3:** Characteristics of patients stratified by platelet count.

	Platelet ≥ 150 × 10^9^/L	Platelet < 150 × 10^9^/L	*p* value
Total number	1602 (56.6%)	1230 (43.4%)	
Age	56.9 ± 12.8	60.2 ± 10.7	<0.001
Gender (M/F)	1313/289	986/244	0.23
Hepatic encephalopathy			0.66
No	1600 (100%)	1227 (99.8%)	
Grades 1-2	2 (0.125%)	3 (0.244%)	
Grades 3-4	0 (0%)	0 (0%)	
Ascites			0.001
No	1536 (96%)	1152 (93.7%)	
Mild	47 (2.93%)	70 (5.69%)	
Moderate	9 (0.562%)	5 (0.407%)	
Gross	10 (0.624%)	3 (0.244%)	
Bilirubin (*µ*mol/L)	12.8 ± 7.67	17.7 ± 11.4	<0.001
Albumin (g/L)	40.0 ± 5.03	38.4 ± 5.28	<0.001
Prothrombin time (sec)	12.3 ± 1.65	13.1 ± 1.65	<0.001
ICG15 (%)	13.0 ± 8.29	21.9 ± 14.9	<0.001
Platelet (×10^9^/L)	231 ± 78.9	103 ± 31.5	<0.001

**Table 4:** Characteristics of patients who underwent major hepatectomy, stratified by eICG15.

	eICG15 ≤ 20.0	eICG15 > 20.0	*p* value
Total number	761 (89.7%)	87 (10.3%)	
Age	55.7 ± 11.7	58.7 ± 10.6	<0.001
Gender M/F (% M)	615/146 (80.8%)	75/12 (86.2%)	0.22
Bilirubin (*µ*mol/L)	10.9 ± 4.49	20.9 ± 12.2	<0.001
Albumin (g/L)	41.2 ± 3.95	34.1 ± 4.40	<0.001
Prothrombin time (sec)	12.1 ± 1.16	13.3 ± 2.44	<0.001
Platelet (×10^9^/L)	209 ± 81	175 ± 67	<0.001
Child-Pugh score			<0.001
5	678 (89.1%)	25 (28.7%)	
6	73 (9.6%)	45 (51.7%)	
7	10 (1.3%)	15 (17.2%)	
8	0 (0.0%)	1 (1.1%)	
9	0 (0.0%)	1 (1.1%)	
ICG15 (%)	10.7 ± 5.59	15.0 ± 7.33	<0.001
Tumour size (cm)	6.97 ± 3.72	8.23 ± 4.42	0.13
Portal venous invasion	42 (5.3%)	10 (1.5%)	0.04
Posthepatectomy liver failure	31 (4.1%)	7 (8.0%)	0.09
In-hospital mortality	28 (3.7%)	7 (8.0%)	0.052
Median overall survival (year)	5.10 ± 0.553	3.01 ± 0.878	0.015
Median disease free survival (year)	1.37 ± 0.215	0.707 ± 0.183	0.018

## Abbreviations

ALP: Alkaline phosphatase
CT: Computed tomography
eICG15: Estimated indocyanine green retention rate at 15 minutes
GGT: Gamma-glutamyl transferase
HCC: Hepatocellular carcinoma
ICG: Indocyanine green
ICG15: Indocyanine green retention rate at 15 minutes
MELD: Model of end-stage liver disease
SPSS: Statistical Product and Service Solution.
